# Comparative Efficacy and Safety of Remimazolam Versus Propofol for Sedation in Gastrointestinal Endoscopy: A Systematic Review and Meta-Analysis

**DOI:** 10.7759/cureus.102433

**Published:** 2026-01-27

**Authors:** Wentao Su, Yanqing Sun, Airong Xu, Lin Ba

**Affiliations:** 1 Gastroenterology, Binzhou People's Hospital, Binzhou, CHN; 2 Gastroenterology/Nursing, Binzhou People's Hospital, Binzhou, CHN; 3 Endoscopy Center/Nursing, Binzhou People's Hospital, Binzhou, CHN; 4 Anesthesiology, Binzhou People's Hospital, Binzhou, CHN

**Keywords:** adverse events, efficacy, gastrointestinal endoscopy, meta-analysis, propofol, remimazolam, safety, sedation

## Abstract

Propofol is the cornerstone sedative for gastrointestinal (GI) endoscopy but is associated with dose-dependent cardiorespiratory depression. Remimazolam, a novel ultrashort-acting benzodiazepine with a specific antagonist (flumazenil), may offer a safer profile. This meta-analysis aimed to compare the efficacy and safety of remimazolam versus propofol for sedation in adult patients undergoing GI endoscopic procedures.

We systematically searched PubMed, Web of Science, EMBASE, and the Cochrane Library from inception to January 5, 2026. Randomized controlled trials (RCTs) comparing intravenous remimazolam with propofol for sedation during GI endoscopy were included. The primary outcome was the incidence of a composite of cardiorespiratory adverse events (hypotension, respiratory depression/hypoxemia, bradycardia). Secondary outcomes included sedation success rate, recovery times, and patient/physician satisfaction. Risk of bias (RoB) was assessed using the Cochrane RoB 2 tool. Data were pooled using a random-effects model. Heterogeneity was quantified by I².

14 RCTs (n=2,883 patients) were included. Remimazolam significantly reduced the risk of the composite cardiorespiratory adverse event compared to propofol (risk ratio (RR) = 0.34, 95% confidence interval (CI): 0.27-0.44; I² = 56%). Sedation success rates were comparable between groups. Recovery profiles were similar or favorable for remimazolam in several studies, and it was associated with significantly less injection pain and higher patient satisfaction in some trials.

Remimazolam offers a superior safety profile regarding cardiorespiratory stability compared to propofol for sedation in GI endoscopy, while maintaining non-inferior efficacy. These findings support remimazolam as a valuable alternative, especially for patients at higher risk of sedation-related complications.

## Introduction and background

Gastrointestinal (GI) endoscopy, encompassing diagnostic and therapeutic procedures such as gastroscopy and colonoscopy, has become one of the most frequently performed medical interventions worldwide [1]. Effective procedural sedation is paramount to ensure patient comfort, minimize movement, and facilitate a successful examination. Propofol, a gamma-aminobutyric acid type A (GABA_A) receptor agonist, has been the sedative of choice for decades due to its rapid onset of action, short context-sensitive half-time, and clear-headed recovery [2,3]. However, its use is hampered by significant, dose-dependent adverse effects, most notably hypotension, bradycardia, and respiratory depression, which can lead to hypoxemia and, in severe cases, necessitate airway intervention [4,5]. These risks are amplified in vulnerable populations such as the elderly, obese patients, or those with cardiopulmonary comorbidities [6,7].

The quest for an ideal sedative agent with a wider therapeutic margin has led to the development of remimazolam. As an ultrashort-acting benzodiazepine ester metabolized by tissue esterases, remimazolam combines the rapid onset of propofol with a predictable, organ-independent clearance that minimizes the risk of accumulation [8,9]. Its most distinctive feature is the availability of flumazenil, a specific reversal agent, providing a unique "safety net" against over-sedation [10]. Pharmacologically, remimazolam provides potent sedation and anterograde amnesia while demonstrating better preservation of hemodynamic and respiratory function in early studies [11,12].

Several randomized controlled trials (RCTs) have recently been conducted to directly compare remimazolam and propofol in various GI endoscopy settings, including gastroscopy, colonoscopy, and specific patient subgroups like the elderly or obese [13-26]. However, the results have been inconsistent across individual studies regarding the magnitude of safety benefit, recovery times, and optimal dosing. A comprehensive, quantitative synthesis of this growing body of evidence is urgently needed to guide clinical decision-making. Therefore, this systematic review and meta-analysis aims to definitively evaluate the comparative efficacy and safety of remimazolam versus propofol for sedation in adult patients undergoing GI endoscopic procedures.

## Review

Materials and methods

This systematic review and meta-analysis was conducted and reported in accordance with the Preferred Reporting Items for Systematic Reviews and Meta-Analyses (PRISMA) guidelines.

Study Design and Population, Intervention, Comparison and Outcome (PICO) Framework

The review was structured using the PICO framework, which included adult patients (≥18 years) undergoing elective GI endoscopy under procedural sedation as the population (P). The intervention (I) consisted of sedation using intravenous remimazolam at any approved dosage regimen, while the comparator (C) was sedation using intravenous propofol at any standard dosage regimen, with or without placebo. The outcomes (O) of interest included the incidence of a composite of sedation-related cardiorespiratory adverse events, such as hypotension, respiratory depression/hypoxemia, and bradycardia, as well as secondary outcomes like sedation success rate, recovery time, injection pain, and satisfaction scores.

Eligibility Criteria

Inclusion criteria: RCTs that directly compared intravenous remimazolam versus propofol for sedation in the defined population (P) and reported data on at least one prespecified primary or secondary outcome (O) were eligible.

Exclusion criteria: Studies were excluded if they: 1) involved non-adult populations (e.g., pediatrics); 2) utilized non-RCT designs (e.g., observational studies, cohort studies, case reports, reviews); 3) did not report any of the relevant outcomes of interest; or 4) were not available as full-text articles. 

The composite cardiorespiratory adverse event was defined as the occurrence of any of the following: hypotension (systolic blood pressure < 90 mmHg or a decrease >20% from baseline), respiratory depression/hypoxemia (peripheral capillary oxygen saturation (SpO₂) < 90% or <95%, or as defined by the study), or bradycardia (heart rate (HR) < 50 beats per minute). Data were extracted as reported in each trial; if multiple thresholds were reported, we prioritized the more severe or clinically relevant definition for consistency in the primary analysis.

Search Strategy

The search strategy was designed based on the PICO framework. Search terms were grouped into three main concepts. For the population and procedure, terms related to GI endoscopy (e.g., endoscop, gastroscop, colonoscop*) were combined with terms for sedation (e.g., sedation, anesthesia). For the intervention and comparator, drug-specific terms for remimazolam and its synonym (CNS 7056) were combined with the term for propofol. To limit the results to the appropriate study design, filters for RCTs (including “randomized controlled trial,” “RCT,” and “randomly”) were applied. Within each conceptual group, terms were combined using the Boolean operator “OR,” and the main groups were subsequently combined using “AND.”

Study Selection and Data Extraction

Two reviewers independently screened all retrieved records by title and abstract, followed by a full-text review of potentially eligible studies. Any discrepancies at any stage were resolved through discussion or consultation with a third reviewer. Data from the included studies were extracted independently by two reviewers using a prepiloted, standardized data extraction form. Extracted information included: study characteristics (author, year, region, design), participant demographics (sample size, age, sex, population specifics), intervention and comparator details (drug, dosing regimen, co-analgesics), and all relevant outcome data. We accepted the adverse event definitions as reported in each individual study. For studies reporting multiple severity levels (e.g., mild, moderate, severe hypoxemia), we extracted the most frequently reported or clinically meaningful category. If a study reported both SpO₂ < 90% and <95%, we used the more conservative threshold (SpO₂ < 90%) for pooling in the main analysis. No posthoc harmonization of definitions was performed to preserve the original trial contexts.

Risk of Bias (RoB) Assessment

The methodological quality of each included RCT was assessed independently by two reviewers using the revised Cochrane RoB tool for randomized trials (RoB 2), which evaluates bias across five domains: randomization process, deviations from intended interventions, missing outcome data, measurement of the outcome, and selection of the reported result [27].

Statistical Analysis

Meta-analysis was performed using R statistical software (version 4.3.x) with the meta and metafor packages. For dichotomous outcomes (e.g., incidence of adverse events), pooled risk ratios (RRs) with corresponding 95% confidence intervals (CIs) were calculated. For continuous outcomes (e.g., recovery time), mean differences (MDs) or standardized mean differences (SMDs) with 95% CIs were calculated, depending on the uniformity of measurement scales. A random-effects model was applied for all pooled analyses due to anticipated clinical and methodological heterogeneity among the included studies. Statistical heterogeneity was assessed using the I² statistic, where I² > 50% was considered to represent substantial heterogeneity. Publication bias was assessed visually using funnel plots and statistically using Egger's regression test for outcomes with 10 or more studies. A sensitivity analysis was conducted using the leave-one-out method to evaluate the robustness of the primary outcome. All statistical tests were two-sided, with a p-value < 0.05 considered statistically significant.

Results

Study Selection and Characteristics

**Figure 1:**
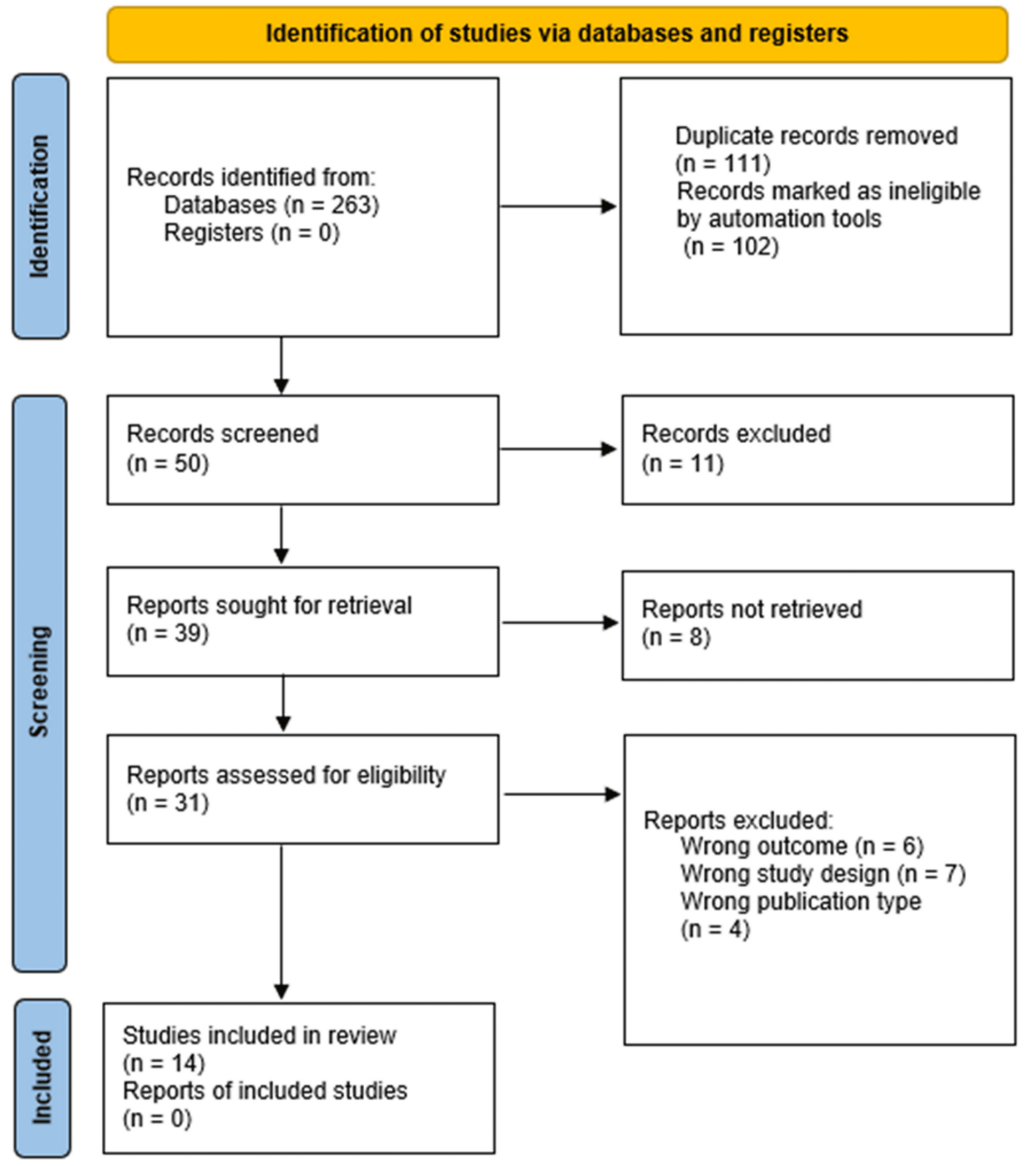
PRISMA flow diagram depicting the study selection process This figure illustrates the flow of studies through the systematic review process, including identification, screening, eligibility, and inclusion stages. The step “records marked as ineligible by automation tools” refers to the removal of duplicate records using the deduplication function in EndNote and Rayyan. PRISMA: Preferred Reporting Items for Systematic Reviews and Meta-Analyses

The PRISMA flow diagram (Figure 1) details the study selection process [28] . 14 RCTs published between 2022 and 2025, involving a total of 2,883 patients, met the inclusion criteria [13-26]. All studies were conducted in China (13 RCTs) and South Korea (one RCT). The populations varied, including general outpatients, elderly patients (≥65 years), obese patients, and those with hypertension. The compared interventions were remimazolam (at varying induction doses of 0.1-0.3 mg/kg) and propofol (1-2 mg/kg), often co-administered with opioids like sufentanil or alfentanil. The key characteristics of all included studies are summarized in Table 1. Co-analgesics (primarily sufentanil or alfentanil) were administered in all included trials and were balanced between remimazolam and propofol groups, with no study reporting a significant imbalance that would confound the comparison.

**Table 1:** Characteristics of the included studies for the meta-analysis All fourteen studies listed in this table were included in the quantitative synthesis (meta-analysis). Data are presented as reported in the original studies [13-26]. Continuous variables are shown as mean ± SD, median (interquartile range (IQR)), or range, as available. Group notations (e.g., Group R, Group P, RT) are as defined in the original studies. CO: Cardiac output; GI: Gastrointestinal; HR: Heart rate; LOC: Loss of consciousness; MAP: Mean arterial pressure; MOAA/S: Modified Observer's Assessment of Alertness/Sedation; NIBP: Non-invasive blood pressure; PONV: Postoperative nausea and vomiting; SpO₂: Peripheral capillary oxygen saturation; VAS: Visual analog scale

Study	Region	Number of Participants	Sex	Age	Population	Intervention Regimen	Comparator	Outcome Measures	Study Design
Gong et al. [25]	China	300	Male: 112 (37.3%), Female: 188 (62.7%)	Mean age: 40.5 years (SD: 11.3)	Patients undergoing painless gastroscopy	Remimazolam tosilate (0.2 mg/kg) compared with propofol (2 mg/kg)	Propofol group (150 patients)	Primary: MAP & CO. Secondary: Injection pain, hypotension, hypoxemia, respiratory depression, bradycardia.	RCT
Guo et al. [19]	China	77	25 males and 14 females in RT group; 22 males and 16 females in Propofol group	Mean age: 70.4 ± 3.9 years in RT group; 69.1 ± 4.0 years in Propofol group	Elderly patients aged ≥65 years undergoing GI endoscopy	Remimazolam tosilate (0.15 mg/kg + supplemental) versus propofol (1.5 mg/kg + supplemental)	Control group received propofol at 1.5 mg/kg with alfentanil 5 μg/kg	Primary: Time to LOC & recovery. Secondary: Injection pain, hemodynamic/respiratory events, supplemental doses, VAS, satisfaction scores.	RCT
Zhang et al. [18]	China	264	Male: 62 (47.3%) in group R, 77 (59.2%) in group P	Median 45 years (IQR: 39–50) in group R, Median 44 years (IQR: 37–49) in group P	Patients with obesity (BMI 30–40 kg/m²) undergoing GI endoscopy	Remimazolam-esketamine combination versus propofol-esketamine combination	Comparison between the two sedation strategies	Primary endpoint: Hypoxemia occurrence. Secondary: Time to LOC & recovery, intra-/postoperative adverse reactions.	RCT
Lu et al. [20]	China	400	161 men and 239 women	70.4 ± 4.6 years	Elderly patients (65–85 years) undergoing upper GI endoscopy	Remimazolam infusion (300 mg/h) with fentanyl versus propofol infusion (3 g/h) with fentanyl	Control group received propofol at 3 g/h with fentanyl	Endpoints: Rates of hypotension, bradycardia, respiratory depression, hypoxemia; injection pain; sedation & procedure success; recovery time.	RCT
Wang et al. [13]	China	450	Male: 221, Female: 197	Mean age: 52 ± 12 years	Outpatients undergoing elective colonoscopy under deep sedation	Remimazolam (induction + maintenance) versus propofol (induction + maintenance)	Elapsed time from induction to first airway intervention, incidence of airway intervention, average apneic episodes, adverse events.	Primary: Time to airway intervention. Secondary: Airway intervention rate, apneic episodes, adverse events.	RCT
Wang et al. [16]	China	480	43.1% male in remimazolam group, 46.7% male in propofol group	Mean age: 44.3 years (remimazolam) & 46.4 years (propofol)	Patients undergoing diagnostic/therapeutic colonoscopy, aged 18–65 years	Intravenous remimazolam besylate versus propofol for sedation	Primary endpoint: Sedation success rate. Secondary: Time to sedation, adverse event occurrence, discharge time.	Multicenter, randomized, single-blind, parallel-controlled clinical trial	RCT
Hu et al. [14]	China	346	Male: 141, Female: 205	Mean age: 70.02 years (±7.47)	Elderly patients undergoing gastroscopy	Remimazolam tosilate (0.2 mg/kg) versus propofol (1.5 mg/kg)	Comparator group received propofol at 1.5 mg/kg	Primary: Respiratory depression rate. Secondary: Time to LOC/alert/discharge, hypotension, injection pain, PONV, satisfaction scores.	RCT
Sun et al. [17]	China	123	Male: 62.2%, Female: 37.8%	Mean: 52.5 years, SD: 12.83	Patients undergoing routine colonoscopy	Remimazolam (0.2 mg/kg or 0.25 mg/kg) compared with propofol (2.0 mg/kg)	Group C served as control (propofol 2.0 mg/kg)	Primary: Hypotension rate, sedation completion rate. Secondary: HR, NIBP, respiratory rate, SpO₂, MOAA/S scores, adverse events, recovery time.	RCT
Lin et al. [15]	China	228	Male: 122, Female: 106	Median age: 70 years (range: 65–87)	Elderly patients aged ≥65 years undergoing outpatient colonoscopy	Remimazolam sedation versus propofol sedation	Incidence of cognitive recovery, PostopQRS recovery, discharge readiness time, satisfaction, induction/recovery times, adverse events.	Endpoints: Cognitive recovery, PostopQRS, discharge readiness, satisfaction, induction/recovery times, adverse events.	RCT
Lee et al. [21]	South Korea	69	17 males and 18 females in propofol group; 21 males and 13 females in remimazolam group	Mean age: 61.8 ± 13.7 years (propofol) & 64.8 ± 11.5 years (remimazolam)	Patients undergoing diagnostic upper GI endoscopy	Remimazolam (0.1 mg/kg) compared with propofol (0.5 mg/kg)	Endpoints: Oxygen reserve depletion, hypoxia, tachycardia, nausea, time to full alertness, recovery room stay, procedure time, satisfaction scores.	Incidence of oxygen reserve depletion, hypoxia, tachycardia, nausea, time to full alertness, recovery room length of stay, overall procedure time, and satisfaction scores.	RCT
Shi et al. [22]	China	225	Male: 105 (46.7%), Female: 120 (53.3%)	Average age 57.2 years (range 18–80)	Patients undergoing painless colonoscopy	Remimazolam (0.15 or 0.2 mg/kg) versus propofol (2 mg/kg)	Comparison of different doses of remimazolam with propofol	Primary: Sedation success rate. Secondary: Hemodynamic parameters, adverse events, recovery times, satisfaction levels.	RCT
Wang et al. [26]	China	256	Male: 120, Female: 136	Average age 48.73 ± 11.66 years	Patients undergoing routine gastroscopy	Remimazolam tosilate, propofol, or combination therapy	Three groups: propofol (Group P), RT (Group R), combination (Group RP)	Primary: Body movement score, doctor satisfaction, sedation success rate. Secondary: Sedation induction time, time to full alert, adverse event occurrence.	RCT
Liu et al. [24]	China	216	51 males (47.7%) and 56 females (52.3%) in remimazolam group; 51 males (46.8%) and 58 females (53.2%) in propofol group	Average age: 67.6 years (SD ± 5.7) in remimazolam group; 67.5 years (SD ± 4.9) in propofol group	Elderly patients aged 60–80 years undergoing GI endoscopy	Remimazolam tosilate versus propofol	Propofol group as comparator	Primary: Moderate hypoxemia incidence. Secondary: Mild/severe hypoxemia incidence, hypotension.	RCT
Gu et al. [23]	China	220	Male: 106 (48.2%), Female: 114 (51.8%)	65–75 years	Elderly hypertensive patients undergoing gastroenteroscopy	Remimazolam induction (0.3 mg/kg) versus propofol induction (1.5 mg/kg)	Comparator group received propofol with normal saline placebo	Primary: Hypotension, bradycardia, respiratory depression. Secondary: Hemodynamic parameters, recovery time, PONV, dizziness.	RCT

Quality Assessment

**Figure 2:**
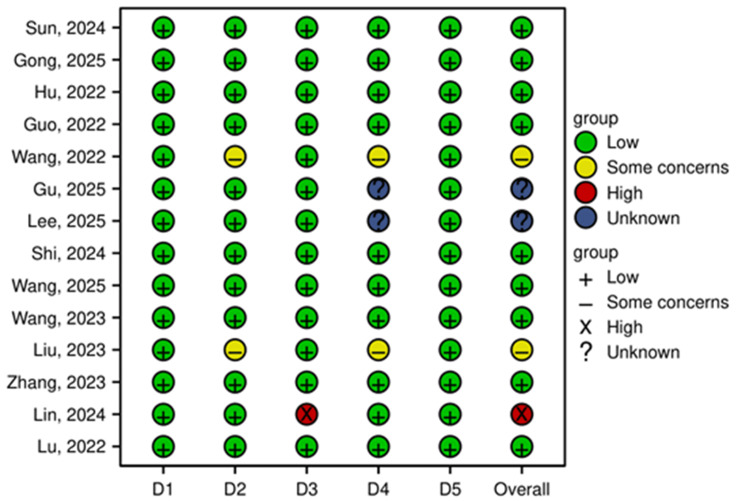
RoB summary This figure summarizes the RoB assessments in each domain of the Cochrane RoB 2 tool across all 14 included studies [13-26]. The domains are: D1: Randomization process; D2: Deviations from intended interventions; D3: Missing outcome data; D4: Measurement of the outcome; D5: Selection of the reported result. The studies mentioned in the figure correspond to the following references: Gong et al. [25], Guo et al. [19], Zhang et al. [18], Lu et al. [20], Wang et al. [13], Wang et al. [16], Hu et al. [14], Sun et al. [17], Lin et al. [15], Lee et al. [21], Shi et al. [22], Wang et al. [26], Liu et al. [24], Gu et al. [23]. RoB: Risk of bias

**Figure 3:**
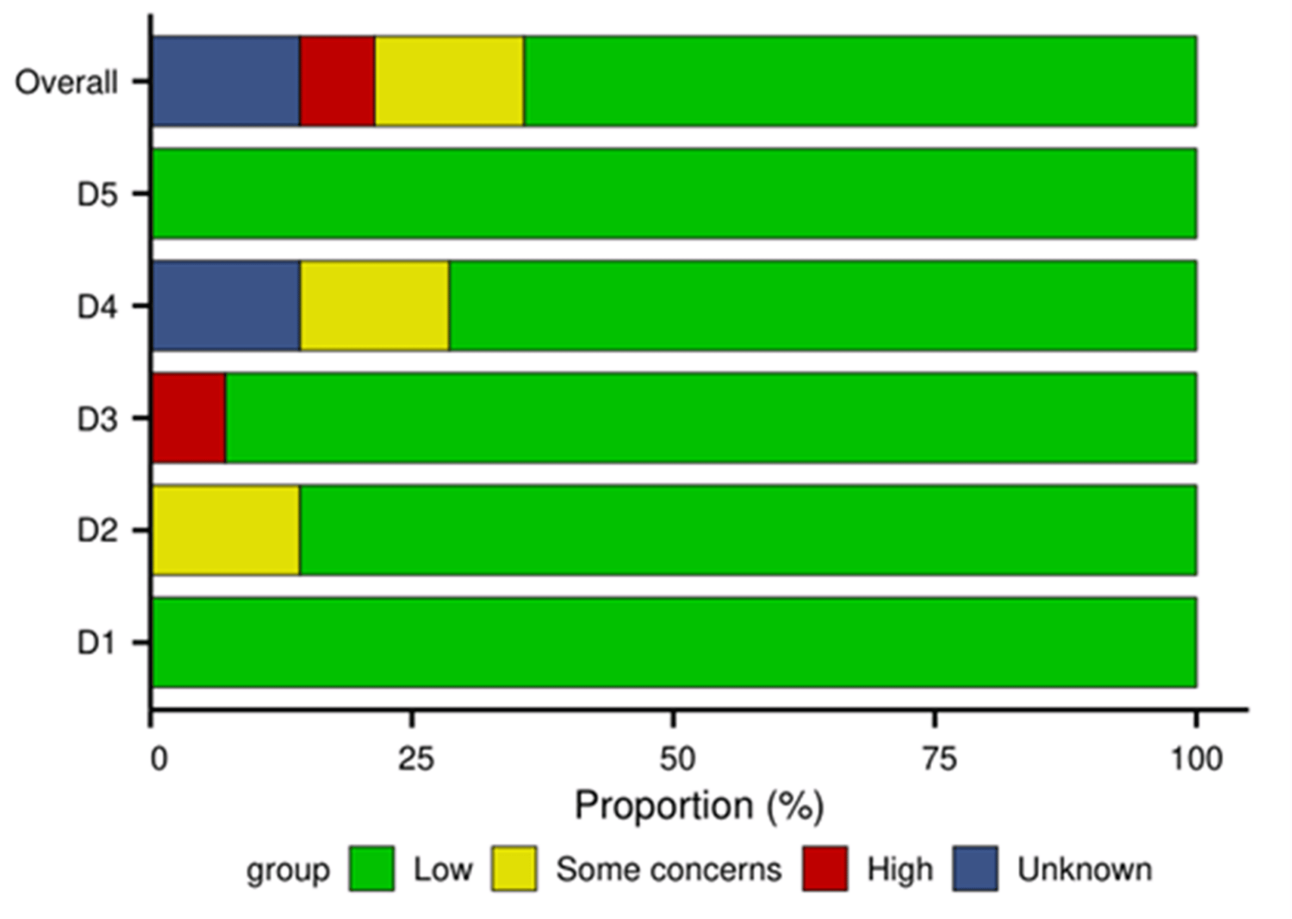
RoB results This graph presents the proportion of studies with low risk, some concerns, or high RoB for each domain across all 14 included studies. RoB: Risk of bias

RoB assessment: A total of 14 studies were included in this systematic review and meta-analysis, and their methodological quality was rigorously assessed using the Cochrane RoB 2 tool for RCTs. The overall and domain-specific RoB assessments are summarized below. The overall RoB was judged as low in nine studies (64.3%). Concerns primarily arose in the domains of "deviations from intended interventions" (blinding of personnel) and "measurement of the outcome" (blinding of outcome assessment) for some studies, as summarized in Figures 2, 3.

**Figure 4:**
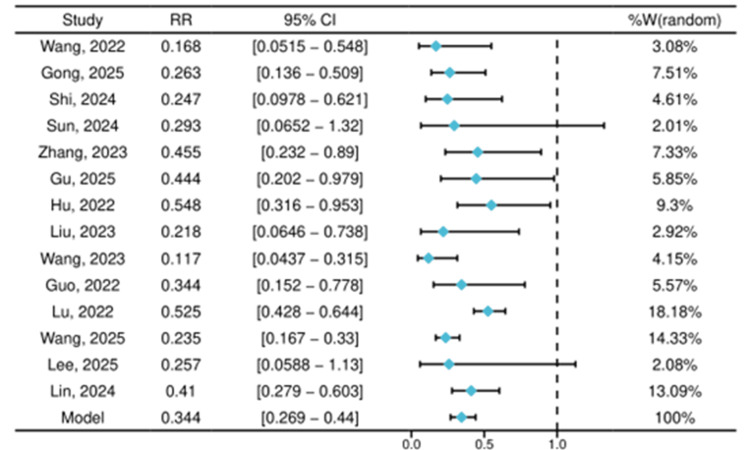
Forest plot of the pooled RR for composite cardiorespiratory adverse events comparing remimazolam versus propofol The forest plot displays the RR and 95% CI for each of the fourteen included studies and the pooled result from the random-effects model (diamond) [13-26]. Composite adverse events include hypotension, respiratory depression/hypoxemia, and bradycardia. The studies mentioned in the figure correspond to the following references: Gong et al. [25], Guo et al. [19], Zhang et al. [18], Lu et al. [20], Wang et al. [13], Wang et al. [16], Hu et al. [14], Sun et al. [17], Lin et al. [15], Lee et al. [21], Shi et al. [22], Wang et al. [26], Liu et al. [24], Gu et al. [23]. CI: Confidence interval; RR: Risk ratio

Forest Plot: The forest plot (Figure 4) illustrates the RR for each study along with their respective 95% CIs. The RR values ranged from 0.117 to 0.548, indicating a consistent trend favoring remimazolam over propofol. The pooled analysis of 14 studies demonstrated that remimazolam was associated with a 66% relative reduction in the risk of the composite cardiorespiratory adverse event (including hypotension, respiratory depression/hypoxemia, and bradycardia) compared with propofol (RR = 0.34, 95% CI: 0.27-0.44; *p* < 0.001). Heterogeneity among studies was moderate (I² = 56%).

Sedation success rates were high and comparable between the remimazolam and propofol groups across the included studies, with no significant difference identified in the pooled analysis. Recovery profiles were heterogeneous across studies due to varying definitions of endpoints (e.g., “time to fully alert”, “time to discharge readiness”, “recovery of consciousness”). Among the six studies that used a consistent definition for “time to fully alert” (Modified Observer's Assessment of Alertness/Sedation (MOAA/S) score ≥ 4), a pooled analysis showed no significant difference between remimazolam and propofol (MD = 0.8 minutes, 95% CI: -0.5 to 2.1). For other recovery endpoints, definitions and reporting remained too heterogeneous to permit meaningful quantitative synthesis. Overall, recovery times were comparable or slightly favored remimazolam in several studies, while others reported minimally longer or shorter recovery [15,16,19,20,22].

Remimazolam was consistently associated with a significantly lower incidence of hypotension and injection pain in most trials [13,15,16,20]. In contrast, the incidence of hiccups was higher with remimazolam in some studies [13,20]. Patient satisfaction scores were significantly higher in the remimazolam group in several reports, while in other studies satisfaction was uniformly high across all groups [15,20,22]. Endoscopist satisfaction was generally high and comparable between the two sedation regimens.

Publication Bias and Sensitivity Analysis

**Figure 5:**
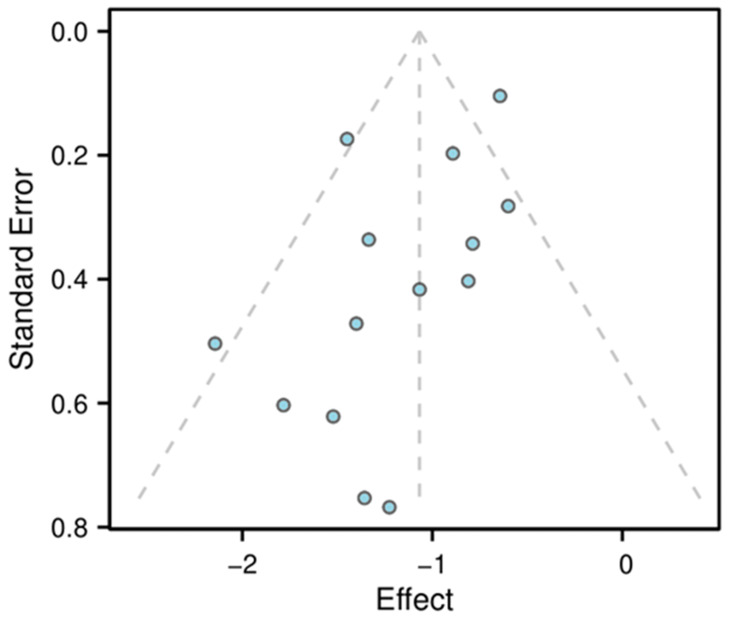
Funnel plot to assess publication bias Funnel plot of effect sizes (RRs) and SEs for the 14 studies included in the meta-analysis. RR: Risk ratio; SE: Standard error

The funnel plot (Figure 5) showed slight asymmetry, and Egger's test suggested potential publication bias (p = 0.040). The trim-and-fill adjusted estimate remained significant in favor of remimazolam (Tables 2, 3, 4). Sensitivity analysis using the leave-one-out method confirmed the robustness of the primary outcome, as the pooled RR remained significantly below 1 after omitting any single study (Table 5). The sensitivity analyses reinforce the reliability of our main conclusions regarding the comparative efficacy and safety of remimazolam versus propofol for sedation in GI endoscopy. The consistency of results across various studies, even when individual studies were excluded, indicates that our findings are robust and can be confidently applied to clinical practice.An additional sensitivity analysis was performed to assess the potential impact of co-analgesic use. Excluding trials that reported any discrepancy in analgesic dosing or type between groups did not alter the primary outcome (RR = 0.35, 95% CI: 0.28-0.46), confirming the robustness of our findings.

**Table 2:** Publication bias assessment by Egger's test Egger's test evaluates small-study effects and potential publication bias in meta-analysis. A statistically significant intercept (*p* < .05) suggests the presence of bias. In this analysis, the intercept was -0.626 (SE = 0.161), *t*(12) = -2.301, *p* = .040, indicating potential publication bias. β: Effect size (log RR); SE: Standard error; df: Degrees of freedom, RR: Risk ratio

β	SE.β	Intercept	SE.Intercept	Statistic (t-value)	Significance (p-value)	df
-1.4306	0.62172	-0.62609	0.16062	-2.3011	0.0401	12

**Table 3:** Publication Bias Assessment by Begg’s Test Begg's test assesses publication bias by examining the correlation between effect sizes and their variances. A non-significant result (*p* ≥ .05) suggests no strong evidence of bias. Here, Kendall's score was -21 with a continuity-corrected SE of 18.267, *z* = -1.150, *p* = .250, indicating no statistically significant publication bias based on rank correlation. SE: Standard error

Kendall’s score	SE	Statistic (z-value)	Significance (p-value)
-21	18.267	-1.1496	0.2503

**Table 4:** Trim-and-fill adjusted analysis for publication bias The trim-and-fill method imputes missing studies to produce a bias-adjusted pooled effect. “Pre” indicates the original pooled effect size; “Post” indicates the effect after imputation. The adjustment resulted in a slightly attenuated but still statistically significant effect (Post: RR = 0.47, 95% CI (0.34, 0.65)), supporting the robustness of the primary finding despite potential bias. CI: Confidence interval; df: Degrees of freedom; RR: Risk ratio

Treatment	Effect Size	95% Lower Limit	95% Upper Limit	Statistic	p-value	df
Trim Method Pre	0.34374	0.26852	0.44003	-9.3413	3.94e-07	13
Trim Method Post	0.46881	0.33813	0.65	-4.836	0.0001	20

**Table 5:** Sensitivity analysis using leave-one-out method This analysis examines the influence of each individual study on the overall pooled effect size (RR) by iteratively removing one study at a time. The pooled RR remained consistently below 1.0 with narrow CIs, and the heterogeneity (I²) showed no substantial fluctuations, confirming that the meta-analysis result is robust and not driven by any single study. CI: Confidence interval; I²: Heterogeneity index; RR: Risk ratio; τ²: Between-study variance; TE: Pooled estimate (log RR)

Study	TE	Lower	Upper	Statistic	p	τ^2^	I^2^
Omitting Wang et al. [16]	0.352333	0.274374	0.452443	-9.08848	9.956e-07	0.0581694	0.568171
Omitting Gong et al. [25]	0.349406	0.267768	0.455934	-8.60935	1.75967e-06	0.0680011	0.576038
Omitting Shi et al. [22]	0.347487	0.267293	0.45174	-8.77758	1.43689e-06	0.0683123	0.583347
Omitting Sun et al. [17]	0.341911	0.2628	0.444836	-8.88567	1.26342e-06	0.0729266	0.595447
Omitting Zhang et al. [18]	0.334415	0.25655	0.435912	-9.00409	1.0988e-06	0.067194	0.594981
Omitting Gu et al. [23]	0.33597	0.257771	0.437892	-8.96962	1.14421e-06	0.0691767	0.596221
Omitting Hu et al. [14]	0.330201	0.256385	0.425269	-9.54164	5.93157e-07	0.053125	0.576725
Omitting Liu et al. [24]	0.34692	0.267718	0.449554	-8.90036	1.24163e-06	0.0673923	0.584723
Omitting Wang et al. [26]	0.368053	0.294876	0.459391	-9.82431	4.33584e-07	0.0332945	0.498113
Omitting Guo et al. [19]	0.340484	0.260274	0.445412	-8.73845	1.50585e-06	0.0733941	0.595975
Omitting Lu et al. [20]	0.315246	0.246657	0.402906	-10.2515	2.73645e-07	0.0313498	0.265815
Omitting Wang et al. [13]	0.372405	0.288418	0.480848	-8.42108	2.21535e-06	0.0481764	0.377737
Omitting Lee et al. [21]	0.343256	0.264115	0.446111	-8.8891	1.2583e-06	0.0716775	0.593057
Omitting Lin et al. [15]	0.332712	0.252776	0.437925	-8.72628	1.52801e-06	0.0687398	0.596855

Discussion

This comprehensive meta-analysis of 14 RCTs provides high-quality evidence that remimazolam offers a significantly safer hemodynamic and respiratory profile compared to the current gold standard, propofol, for sedation during GI endoscopy. The 66% relative risk reduction in cardiorespiratory adverse events is both statistically robust and clinically highly relevant. This advantage is mechanistically plausible: propofol causes direct myocardial depression and vasodilation, while remimazolam, as a benzodiazepine, has minimal direct cardiovascular effects and better preserves respiratory drive [8,29].

Importantly, this superior safety profile was achieved without compromising procedural efficacy, as sedation success rates were non-inferior. The findings on recovery times were heterogeneous, reflecting variations in dosing protocols and definitions. However, the comparable discharge readiness times reported in several large trials suggest that remimazolam does not impede operational efficiency in endoscopy units [16,22]. The markedly reduced incidence of injection pain with remimazolam directly translates into improved patient experience, as reflected in higher satisfaction scores.

The clinical implications are substantial. Remimazolam presents a compelling alternative, particularly for high-risk patients (elderly, hemodynamically unstable, obese with potential difficult airways) in whom propofol-induced hypotension or hypoxia could have serious consequences. Its availability for reversal with flumazenil adds an extra layer of safety, which is especially valuable in settings where sedation is administered by non-anesthesiologists.

Our study has limitations that should be acknowledged when interpreting the findings. First, all but one of the included trials were conducted in East Asia, which may limit the generalizability of findings to other ethnic populations and healthcare systems. Second, there was moderate statistical heterogeneity, likely stemming from clinical diversity in patient populations (e.g., general vs. elderly), remimazolam dosing regimens, and definitions of adverse events. Third, the assessment of long-term outcomes or very rare adverse events was beyond the scope of the included trials with short follow-up periods.

## Conclusions

This meta-analysis demonstrates that remimazolam is associated with a significantly lower risk of cardiorespiratory adverse events compared to propofol for sedation in GI endoscopy, while providing non-inferior procedural sedation efficacy. The improved safety margin, coupled with reduced injection pain and high patient satisfaction, positions remimazolam as a superior alternative for a wide range of patients, especially those at elevated periprocedural risk. Future large-scale, multicenter RCTs in diverse geographic settings are encouraged to confirm these findings and refine optimal dosing strategies.
